# Tethered Cord Syndrome: Role of Imaging Findings in Surgical Decision-Making

**DOI:** 10.7759/cureus.44854

**Published:** 2023-09-07

**Authors:** Nolan Otto, Jennifer Kucera, Laura L Hayes, Tushar Chandra

**Affiliations:** 1 Radiology, University of Central Florida, College of Medicine, Orlando, USA; 2 Pediatric Radiology, Nemours Children's Hospital, Orlando, USA

**Keywords:** voiding cystourethrogram, magnetic resonance imaging, ultrasound, vesicoureteral reflux, neurogenic bladder, spinal dysraphism, conus medullaris, filum terminale, tethered cord syndrome

## Abstract

For infants presenting with urinary problems or lower extremity weakness, imaging is ordered to investigate spinal pathology. Tethered cord syndrome (TCS) often manifests without conclusive anatomic evidence. In our case, a premature infant presented with urosepsis and was found to have an asymmetric gluteal crease and a sacral dimple. Renal ultrasound showed mild hydronephrosis, and a cystourethrogram revealed bilateral high-grade vesicoureteral reflux. Ultrasound and magnetic resonance imaging demonstrated a borderline low-lying spinal cord at the mid-L3 vertebral level. Urodynamic testing to confirm neurogenic bladder could not be completed on the first attempt due to urinary tract infection and on the second attempt due to instrument intolerance. Despite the lack of conclusive imaging evidence of a tethered cord, enough supportive clinical data was present to proceed with surgical intervention with the goal of preventing the progression of neurological dysfunction. Because TCS is ultimately a clinical diagnosis, appropriate management should not be discouraged by inconclusive or borderline imaging findings.

## Introduction

Imaging is frequently requested for patients presenting with symptoms concerning tethered cord syndrome (TCS) [1]. Magnetic resonance imaging (MRI) is the gold standard for evaluating neural anatomy in all patients. Infants have partially ossified vertebrae and thus may be screened with ultrasound. Classically, a tethered cord presents with symptoms including lumbosacral pain, lower extremity weakness, and neurogenic bladder dysfunction, as well as imaging findings of a low-lying, dorsally positioned conus medullaris with limited dependent movement [2]. However, symptomatic patients may have normal anatomy, and the presence of abnormal anatomy on imaging does not always produce symptoms of a tethered cord. Also, patients with typical symptoms, albeit inconclusive imaging, have undergone standard surgical treatment with significant relief of symptoms [3]. The importance of integrating clinical and radiological data for the diagnosis of TCS is demonstrated in our case.

## Case presentation

A 14-day-old male in the neonatal intensive care unit for supportive care due to prematurity had a new onset of bradycardia and desaturation events. The patient was subsequently diagnosed with E. coli urosepsis. Birth history included a cesarean section at 32 weeks and one day due to fetal bradycardia and the mother’s preeclampsia with severe features. After appropriate care and completion of antibiotic therapy, the patient presented at 29 days of age to investigate the etiology of a urinary tract infection.

Vital signs were within normal limits. A physical exam showed a bifid gluteal crease and a sacral dimple but was otherwise normal with intact lower extremities and without abnormal muscle tone or focal neurological deficits. Blood cell counts showed no signs of residual infection. Serum chemistries were normal, including a blood urea nitrogen (BUN) and a creatinine of 4.0 and 0.3, respectively. A urinalysis with microscopy was negative for blood, protein, nitrites, leukocyte esterase, or white cells.

**Figure 1:**
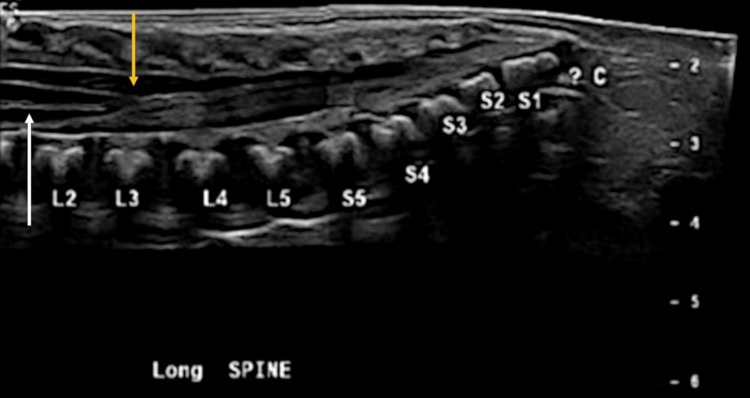
Spinal ultrasound Longitudinal spinal ultrasound demonstrates a low-lying cord with the tip of the conus medullaris non-dependently positioned at the middle of the L3 vertebral level (yellow arrow). Incidental finding of a split central echogenic complex likely represents transient dilatation of the central canal (i.e., ventriculus terminalis) (white arrow). Radiological images obtained by the authors.

**Figure 2:**
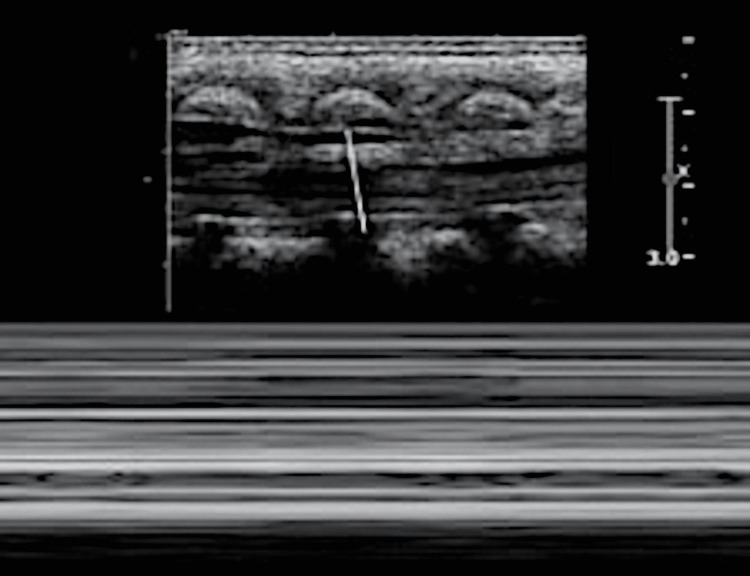
Spinal ultrasound M-mode captured decreased dependent motion of the cauda equina. Radiological images obtained by the authors.

**Figure 3:**
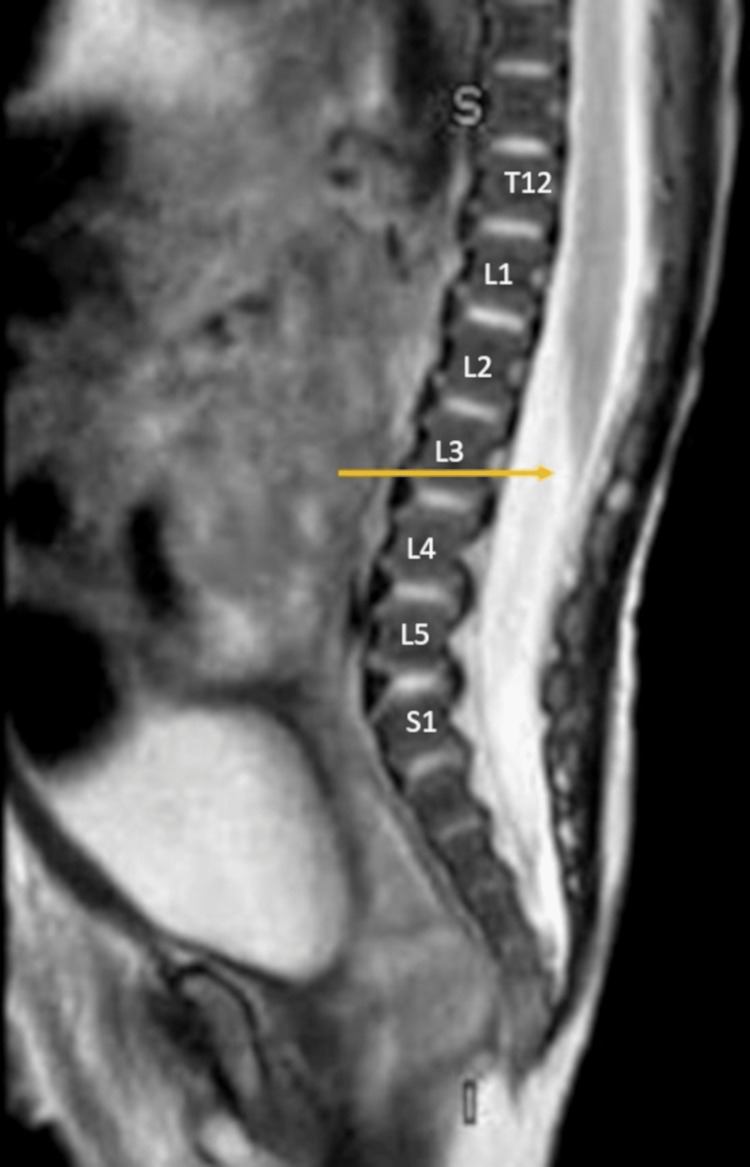
MRI Sagittal T2-weighted MRI sequence confirming borderline low-lying cord with the tip of the conus medullaris terminating at the middle of the L3 vertebrae (yellow arrow). Radiological images obtained by the authors.

The presence of an abnormal gluteal crease and sacral dimple raised concerns about a possible underlying spinal dysraphism, so a spinal ultrasound was performed. This demonstrated the conus medullaris being non-dependently positioned at mid-L3 vertebral level with reduced cauda equina motion (Figure 1, 2). An MRI confirmed borderline low-lying cord terminating at mid-L3 (Figure 3), but no other anatomic abnormalities of occult spinal dysraphism.

**Figure 4:**
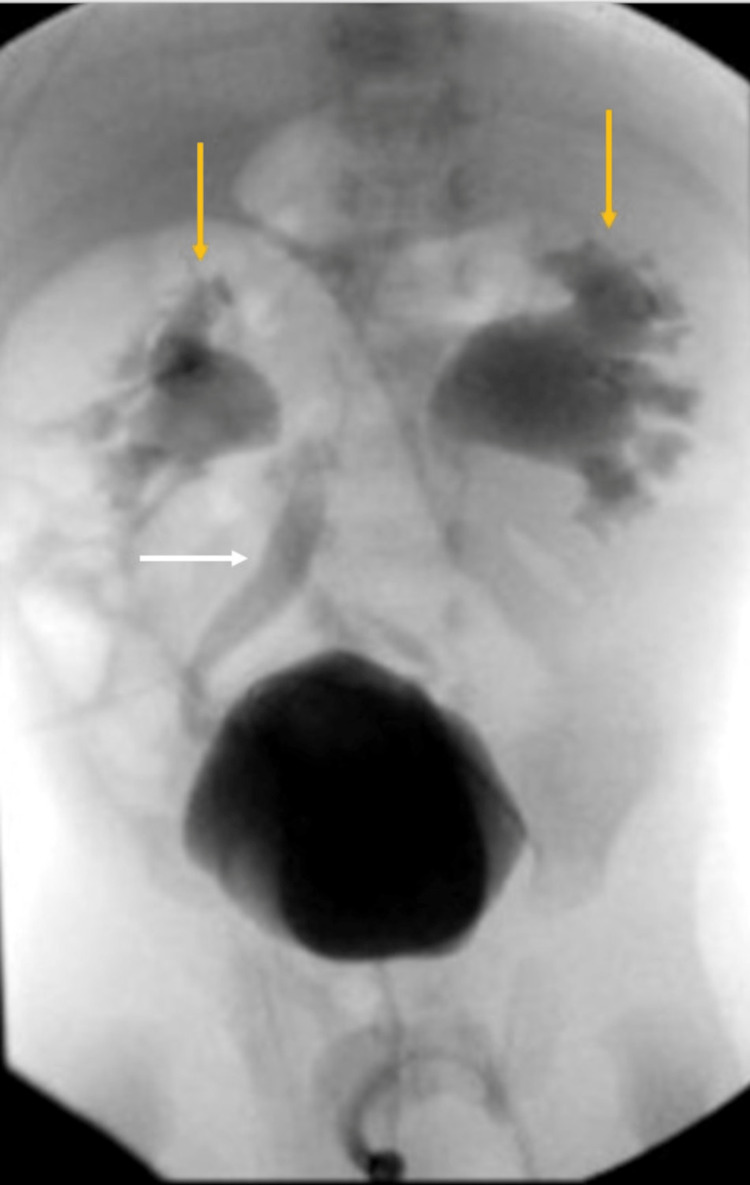
Voiding cystourethrogram Voiding cystourethrogram demonstrating bilateral grade 4 vesicoureteral reflux. There is mild dilatation of the right ureter (white arrow) and marked dilatation of the renal collecting systems with calyceal blunting bilaterally (yellow arrows). Radiological images obtained by the authors.

To investigate the urinary tract for structural abnormalities or complications of infection, a renal ultrasound was performed. This demonstrated bilateral mild pelviectasis, central and peripheral caliectasis, and mild dilatation of the distal ureters. A voiding cystourethrogram demonstrated bilateral high-grade vesicoureteral reflux with moderate post-void residual bladder volume. There was no evidence of ureteral ectopia or posterior urethral valves (Figure 4).

Although not conclusive, the spinal imaging provided enough evidence to suggest the possibility of TCS as a neurological cause of vesicoureteral reflux. Also, the voiding cystourethrogram showed no obvious evidence of anatomic abnormalities to suggest a structural etiology. Thus, neurogenic bladder or bladder-sphincter dyssynergia was most likely. Prior to initiating a treatment plan, urodynamic testing was attempted to confirm neurological dysfunction but could not be completed. A first attempt was aborted due to the discovery of a urinary tract infection, and a second attempt failed due to rectal catheter intolerance due to the patient resisting the catheter and continued bouts of stool.

Thus, a determination between neurogenic versus anatomic etiology for reflux could not be definitively made; however, given the clinical presentation and imaging findings suggestive of TCS, prompt surgical intervention was recommended to prevent the possible progression of neurological deficits. At age three months, the patient underwent laminectomy with release of the filum terminale without complications.

At age five months, post-surgical urodynamic studies demonstrated detrusor-sphincter dyssynergia without detrusor overactivity. This confirmed the presence of neurological dysfunction as the most likely cause of vesicoureteral reflux, consistent with TCS. Also, repeat voiding cystourethrograms demonstrated non-progression of right-sided grade 4 hydronephrosis and improvement of left-sided hydronephrosis to grade 3, confirming stabilization of the patient's condition.

## Discussion

TCS occurs when the filum terminale is excessively tense, causing stretch-induced ischemia of the neural tissues at the caudal end of the spinal cord [1]. Cord tethering can result from congenital spinal dysraphisms or can be acquired from local malignancy, trauma, or post-surgical complication. TCS is diagnosed primarily by clinical criteria, although imaging evidence can assist in the diagnosis.

The filum terminale is an extension of the spinal cord that attaches the conus medullaris to the periosteum of the coccyx. The proximal third is similar to the conus medullaris and contains nervous and glial cells. The more distal portions progressively transition to fibrous connective tissue [2]. The function of a normal filum terminale is to protect the spinal cord within the vertebral canal by fixing it at a particular level and limiting, but not restricting, lateral movement. In most healthy infants, children, and adults, the conus terminates at the L1-L2 intervertebral disc level [3]. The conus medularis is below the L1-L2 level in all fetuses less than 18 weeks gestational age and progressively ascends in the vertebral canal throughout gestation [4]. Regardless of gestational age, the conus medullaris terminates below the adult level in 13% of neonates less than 30 days old and in 4.7% of neonates around 61-100 days [5,6]. A borderline low-lying cord is considered to terminate between the L2-L3 disc space and the mid-L3 vertebral level [7]. The finding, however, may be equivocal and require more evidence to exclude disease. A cord terminating below the L3 vertebral level is considered low-lying and more indicative of congenital disease [7].

Tethered cord can be acquired or congenital. Acquired tethered cord is more common in the adult population. The filum terminale can be displaced by locally invasive malignancies or fixed to adjacent structures by intradural fibrous scars. Scarring is often acquired from vertebral trauma or as a post-surgical complication. Congenital tethered cord is caused by spinal dysraphisms, and the clinical presentation has a variable rate of onset. Open spinal dysraphisms are apparent on gross physical examination; investigation for closed spinal dysraphisms is initiated after observing neurological consequences or skin signs (e.g., split gluteal crease or sacral dimple, as in this case) [8]. It is rare for simple sacral dimples in asymptomatic infants to be associated with spinal malformations; however, atypical sacral dimples are a high-risk factor and should therefore be investigated [9,10]. Congenital TCS may present as early as infancy. Pediatric patients can have a gradual onset of symptoms as the conus is progressively placed under greater tension as their spines extend during development [11]. Adult patients may be asymptomatic until an event or degenerative changes exacerbate tension on the filum terminale [12].

Malformations and inelastic structures abutting or originating from the filum terminale can, but not always, cause tension. Forces pulling on the conus medullaris cause it to stretch, which limits perfusion. Over time, this stretch-induced ischemic damage causes neurological deficits in the distribution of the lumbosacral spinal nerves [1].

The symptoms of TCS include pain and neurological dysfunction. Pain is non-dermatomal and in the lumbosacral region, perineum, or lower extremities. Especially in infant populations, the predominant presenting symptom is neurological dysfunction. Lower motor neuron-type neuropathy of the lower extremities can result in weakness and gait abnormalities. Autonomic dysfunction often manifests as neurogenic bladder or bladder-sphincter dyssynergia. Patients may then have urinary tract infections due to vesicoureteral reflux or progressive incontinence [12].

In pediatric populations, the posterior elements of the spine do not fuse until approximately six months of age, allowing ultrasound to be a useful first exam [13]. Sonographic evidence of a tethered cord includes caudal positioning of the conus medullaris below the L2/L3 vertebral level as well as reduced motion of the conus or cauda equina [14, 15]. MRI can confirm the positioning of the conus [3]. The primary role of MRI in a suspected case of TCS is to delineate the exact level of cord termination and rule out additional abnormalities of the lower end of the cord. Additional imaging with a sagittal T2 sequence acquired with the patient in a prone position can be useful to evaluate for mobility of the lower end of the cord within the spinal canal and can provide additional information in borderline cases.

Imaging is also useful for characterizing neurological complications of TCS. Patients with high-grade vesicoureteral reflux may have a renal ultrasound showing pelvic and calyceal dilation. Recurrent urinary tract infections can lead to hyperechoic, scarred kidneys with parenchymal thinning. A voiding cystourethrogram demonstrates the severity of vesicoureteral reflux but does not absolutely differentiate between anatomic and neurologic etiologies. Urodynamic testing is needed to confirm neurogenic bladder or bladder-sphincter dyssynergia [16].

Standard treatment is surgical, with sectioning of the filum terminale and removal of any fixating abnormal anatomy. The goals of treatment are the improvement of pain and the return of, or at least stabilization of, neurological function. Prophylactic surgery is an option for patients who are asymptomatic yet have an elongated, thickened, or fatty filum terminale. Such patients are at risk for irreversible dysfunction once symptoms present [17]. One study of 144 children with TCS due to thickened or fatty filum terminale manifesting as symptomatic bowel and bladder dysfunction demonstrated improvement or stabilization in 80% of patients following cord release [18]. Of 64 children treated with prophylactic surgery for thickened or fatty filum, none had a new onset of symptoms after a mean of 6.6 years of follow-up [18]. Outcomes are superior for children when operated on at less than one year of age; older children with deficits are less likely to recover [19]. As many as 30% of patients may require a second, third, or fourth untethering after the initial surgery [20].

Although imaging can provide anatomical and physiological context, the diagnosis and management of TCS are ultimately clinical. In the absence of imaging studies that provide conclusive findings, patients presenting with typical pain and neurological symptoms should still receive the appropriate diagnosis and surgical evaluation.

## Conclusions

TCS is primarily diagnosed by clinical presentation, although imaging data is useful for defining the etiology and assessing the severity of sequelae. The majority of patients benefit from surgical release of a tethered cord, resulting in prevention or improvement of symptoms, although some require repeated procedures. In this case, a patient was appropriately diagnosed and treated for TCS and had stabilization of symptoms. Patients with typical clinical symptoms of TCS should receive appropriate treatment, even in cases of indeterminate imaging criteria.
